# A Zinc Oxide Interconnected Hydroxypropyl-Beta-Cyclodextrin/rGO Nanocomposite as an Electrocatalyst for Melatonin Detection: An Ultra-Sensitive Electrochemical Sensor

**DOI:** 10.3390/s25113266

**Published:** 2025-05-22

**Authors:** Kuo-Yuan Hwa, Aravindan Santhan, Chun-Wei Ou, Cheng-Han Wang

**Affiliations:** 1 Department of Molecular Science and Engineering, National Taipei University of Technology, Taipei 10608, Taiwan; aravindan@mail.ntut.edu.tw; 2 Graduate Institute of Organic and Polymeric Materials, National Taipei University of Technology, Taipei 10608, Taiwan; t112518088@ntut.edu.tw (C.-W.O.); t112518009@ntut.edu.tw (C.-H.W.)

**Keywords:** melatonin, biological samples, electrochemical sensing, zinc oxide flaky structures, hydroxypropyl-beta-cyclodextrin@rGO

## Abstract

Nanocomposite hydroxypropyl-beta-cyclodextrin functionalized reduced graphene oxide sheets (HpβCD@rGOs) with zinc oxide flaky structures (ZnOFs) were synthesized. The ZnOFs/HpβCD@rGOs were first characterized to examine their physicochemical characteristics. The ZnOFs exhibited a highly crystalline structure intertwined with HpβCD@rGO sheets. The electrocatalyst experienced excellent electrochemical oxidation current responses toward melatonin (MTN). The interaction between the catalyst and MTN improves electrochemical activity through a synergistic action, which can be measured by a glassy carbon electrode (GCE) modified with ZnOFs/HpβCD@rGOs. This modified electrode with the increased reactive sites and a large electrochemically active surface area allows the rapid oxidation reaction of MTN. The oxidation of MTN was detected and measured with a linearity range around 0.014–0.149 and 1.149–643.341 (µM), with a low detection limit (LOD) of around 0.0105 µM or 10.5 nM. The sensitivity was around 6.19 μA μM^−1^ cm^−2^. The constructed electrode demonstrated a notable level of selectivity to MTN when the interfering (biological) chemicals with a similar structure to MTN were introduced. The real samples were tested in order to examine whether the ZnOFs/HpβCD@rGOs/GCE can be developed for the biomedical monitoring of compounds. The results suggest that ZnOFs/HpβCD@rGOs/GCE can detect MTN in in vitro human samples. Furthermore, the cost-effectiveness, enhanced electrochemical capabilities, and easy fabrication of the electrode make the ZnOFs/HpβCD@rGOs composite a feasible solution for the future industrial development of monitoring tools as sensors.

## 1. Introduction

The existence of melatonin (MTN), a hormone that is produced naturally from the pineal gland in the brain, is known for the monitoring and regulation of the circadian cycles of human life [1]. MTN is an indoleamine hormone produced in the vertebrate pineal gland, located in the epithalamus [2]. It pertains to and influences our everyday sleep–wake process, as well as numerous activities related to the immune structure, the cardiovascular structure, cell regulations, retinal physiological processes, and reproduction [3,4]. Besides these actions, it has antioxidant, anti-inflammatory, anti-tumor, and free-radical scavenging functions [5]. The aforementioned physiological processes are facilitated by the connection of melatonin receptors. The MTN is generated and secreted in vertebrates by pinealocytes located at the pineal gland and locally in photoreceptor cells present in the retina [6]. It affects the circadian rhythm, which is associated with both light and dark conditions [7]. MTN can be found at concentrations ranging from 10 to 200 pgmL^−1^ in light to dark conditions [8,9].

The lower levels of MTN are associated with neurodegenerative disorders like Alzheimer’s, Parkinson’s disease, and possibly Ebola virus infections [10]. Moreover, MTN is known to regulate metabolic processes, including glucose homeostasis and energy metabolism; regulates numerous other physiological functions; and also inhibits pigment metabolism [10]. In addition, MTN is possibly able to cure serious brain injuries because of its stimulatory effect and high diffusion rate across the barrier between the blood and the brain [11]. The primary component in supplements in opposition to short-term sleep disorders consists of MTN, which is being shown to be more important. MTN is found in tissues and bodily fluids, including saliva, blood, and urine [5,12]. As a result, MTN monitoring is essential due to the apprehension concerning the impact on the hormone systems and other metabolic processes [5,7].

Various methods for detection are accessible and employed, including micellar electrokinetic chromatography (MEC), colorimetric (CM), electrochemical sensing (ES), radioimmunoassay (RIA), chemiluminescence (CL), spectrofluorometric (SF), and numerous others [1,13,14,15]. Electrochemical methods have several advantages that include low cost, ease of use, high sensitivity, and minimal workforce requirements. However, the other approaches described above are hindered by numerous drawbacks, including the need for extensive workers, high expenses, the excessive waste of samples, and the difficulty of preparing samples. The proposed electrocatalyst is an essential component in the detection of MTN, for which the electrochemical approach was developed. Electrocatalysts are investigated for a variety of targeted analytes. But challenges occur when it comes to developing a low-cost electrode material that exhibits excellent electrical conductivity, with an uninterrupted and stable response, when studied for stability tests and other repeatability and reproducibility tests [5,7,16,17].

Metal oxides, which are a type of inorganic material, consist of affordable and widely available components. They are considered a significant group of useful materials. When compared to other forms of metal compounds, metal oxides have a unique benefit due to their wide range of compositions and structures. This allows for flexibility in electronics and crystal structures, resulting in a variety of desired chemical and physical characteristics. Metal-oxide-based compounds have attracted significant attention for a wide range of applications because of their compositional/structural diversity, adjustable tunability, inexpensiveness, abundance in the Earth’s crust, simple synthesis, and sustainability [18]. Some of those metal oxides are zinc oxide, copper oxide, nickel oxide, iron oxide, tungsten oxide, molybdenum oxide, manganese oxide, cadmium oxide, and silver oxide. Zinc oxide (ZnO) is attracting significant interest among other metal oxide materials because of its notable characteristics, including its affordability, abundance, binding energy of 60 meV, and broad bandgap of 3.37 eV [19,20,21]. ZnO is classified as a direct and broad bandgap metal oxide semiconductor material. Furthermore, ZnO has found extensive use in various technologies like supercapacitors, sensors, photocatalysis, and solar cells [22,23,24,25].

Carbon-based materials are elements characterized by a significant amount of carbon in their chemical structure. The construction, characteristics, and potential uses of these compounds differ considerably, according to the configuration and order of carbon atoms. Some of such carbonaceous materials are graphene, fullerenes, activated carbon, carbon nanotubes, carbon fibers, carbon black, and coal [26,27,28]. Graphene has recently gained attention as a highly fascinating material composed of carbon atoms (sp^2^ hybridization) structured in a honeycomb-like arrangement with a layer thickness approximately equivalent to that of one individual carbon atom [29]. It exhibits exceptional transparency and crystalline structure, along with remarkable electrical characteristics. Graphene exhibits distinctive mechanical and electrical characteristics as a result of its stronger σ bonds and delocalized π electrons by the unhybridized p orbitals, respectively. Moreover, it has favorable thermal characteristics in addition to having excellent mobility of electrons. The structure of graphene oxide (GO) closely resembles that of graphene. The only distinction that exists is in the presence of oxygen-carrying functional groups in GO, such as epoxide (C-O-C), carboxyl (C-OOH), hydroxyl (C-OH), carbonyl (C=O), and others [26,30]. These groups primarily exist on the outer and basal surfaces and are added during the production of graphene oxide from graphite flakes. GO’s remarkable colloidal stability in water, as well as its distinctive optical and mechanical capabilities, are a result of these functional categories.

Reduced graphene oxide (rGO) exhibits features that are analogous to graphene and serves as an intermediary between graphene and graphene oxide (GO) [31]. The existence of defects and oxidized chemical compounds in rGO results in mechanical, optical, and conductivity capabilities that are comparable to those of pure graphene [32]. It exhibits superior conductivity and may be readily prepared in the desired quantity. The thermal properties of reduced graphene oxide (rGO) are exceptionally high, with a small 11% overall weight loss observed up to a temperature of 800 °C [29]. Moreover, rGOs exhibit exceptional catalytic properties that are significant in various areas such as supercapacitors, batteries, photocatalysts, bio-compatibility-related studies, and electrochemical investigations [33]. Overall, this material contains outstanding characteristics, making it highly suitable for various electrochemical applications [34,35,36,37].

In addition to these, cyclodextrins are oligosaccharides composed of 6 to 8 units of glucose linked by 1,4-glucosidic linkages [38]. They are classified as alpha (α), beta (β), and gamma (γ)-cyclodextrins. β-cyclodextrin (βCD), within this category of cyclodextrins, has significant chemical selectivity toward particular compounds. It forms long-lasting host–guest combination complexes with high binding values that exceed 10^3^. Also, it is important to note that βCD has excellent solubility in water and plays a significant role in keeping rGOs soluble and stable, hence ensuring its environmental suitability [38,39]. Oligosaccharide compounds have a hydrophobic center and a hydrophilic outside layer that contains a high concentration of hydroxyl groups [40]. This unique structural configuration facilitates targeted interactions between the host and guest, hence improving the material’s ability to selectively attach to desired molecules from guests to create complex connections within the surface’s cavity [41]. Hydroxypropyl-beta-cyclodextrin (HpβCD) is a structurally modified form of beta-cyclodextrin (βCD). HpβCD is chemically modified by attaching hydroxypropyl category to the hydroxyl groups of the glucose components of βCD. This alteration improves the solubility of HpβCD in both water and other solvents as compared with native βCD, hence increasing its versatility for a range of applications [38].

The objective of the present study is to develop a precise and accurate method for detecting melatonin (MTN) using an electrocatalytic approach with a glassy carbon electrode (GCE) modified with ZnOFs/HpβCD@rGOs. The ZnOFs/HpβCD@rGOs composite was analyzed using XRD, FTIR, Raman, FE-SEM, EDX, and TEM. Based on our knowledge and the previously published papers, this strategy is being presented for the first time, demonstrating excellent sensitivity and selectivity, with a high level of detection down to the nanomolar range, for the identification of melatonin in biological fluids.

## 2. Chemicals and Techniques

For the synthesis of hydroxypropyl β-cyclodextrin with reduced graphene oxide HpβCD@rGOs, zinc oxide (ZnOFs), and ZnOFs/HpβCD@rGOs, materials including graphene oxide, L-ascorbic acid, hydroxypropyl-β-cyclodextrin, sodium hydroxide, zinc nitrate hexahydrate, graphite, and potassium permanganate were all utilized. For the electrochemical study, melatonin and selectivity compounds like serotonin, epinephrine, dopamine, nicotinamide adenine dinucleotide (NADH), tryptophan, glutathione, creatinine, glucose, and ascorbic acid were used. Multiple buffer solutions were prepared using disodium hydrogen phosphate and sodium dihydrogen phosphate. Sodium hydroxide and hydrochloric acid were employed to manipulate the various pH levels. Each of the abovementioned chemical components was acquired from Taiwan’s Sigma Aldrich in the form of laboratory standards.

The X-ray diffraction (XRD) technique was used to study the structure and analytical properties of ZnOFs/HpβCD@rGOs. The XRD analysis was performed using a PANanalytical diffractometer (PANalytical, Worcestershire, UK) equipped with Cu Kα radiation (k = 1.54 Å). The Fourier transform infrared spectroscopy technique was examined using an FT-IR instrument, namely the Perkin Elmer CHI1000C model 6600 (Springfield, IL, USA). The morphological examination and the energy dispersive X-ray (EDX) spectrum of the samples were conducted and analyzed using a Hitachi SU6600 Field-Emission Scanning Electron Microscope (FE-SEM, Tokyo, Japan). A pH meter with high accuracy (pH 500) was employed, along with a pH glass electrode, to test the pH of the buffer solution that was prepared for electrochemical analysis. An electrochemical investigation was conducted utilizing a CHI 6111E workstation and a typical three-electrode system. The working electrode was a glassy carbon electrode (GCE), the reference electrode was a saturated Ag/AgCl, and the counter electrode was a platinum wire.

### 2.1. Preparation of ZnOFs, HpβCD@rGOs, and ZnOFs/HpβCD@rGOs

The ZnOFs, HpβCD@rGOs, and ZnOFs/HpβCD@rGOs were synthesized using a simple method involving ultrasonication and hydrothermal treatment [42,43]. The synthesis processes for ZnOFs/HpβCD@rGOs, HpβCD@rGOs, and ZnOFs are detailed in this section. To develop hydroxypropyl-beta-cyclodextrin with reduced graphene oxide (HpβCD@rGOs), a specific amount of graphene oxide (GO) was reduced further following the procedure detailed below. About 0.5 g of graphene oxide (GO) was added to double-distilled (DD) water and subjected to ultrasonic processing for 2 h. The hydroxypropyl-beta-cyclodextrin (0.3 g) and the L-ascorbic acid (0.9 g) mixtures were added to the previous mixture and continuously combined while sonicating for 4 h and stirring for 2 h. The product obtained was thereafter carried to an autoclave and subjected to hydrothermal processing at a temperature of 120 °C for 12 h. When the temperature reached room temperature, the final product was rinsed with distilled water and ethanol to eliminate any undesired substances. 

Zinc oxide was developed using the ultrasonication approach, with some adjustments, based on an earlier investigation published by [44]. The main components were 0.2 M of zinc nitrate hexahydrate dissolved in 70 mL of distilled water and then mixed with constant stirring. In addition, sodium hydroxide was used as a surfactant and combined with the aforementioned solution while stirring. Following 4 h of stirring, a more concentrated white mixture was acquired, which was next treated with 100 Hz for 8 h of ultrasonication. The ZnO sample was then centrifuged using DI water and ethanol, repeating the process multiple times to eliminate the impurities. In the end, the material becomes dry by being put in a hot oven at a temperature of 50 °C overnight.

**Scheme 1:**
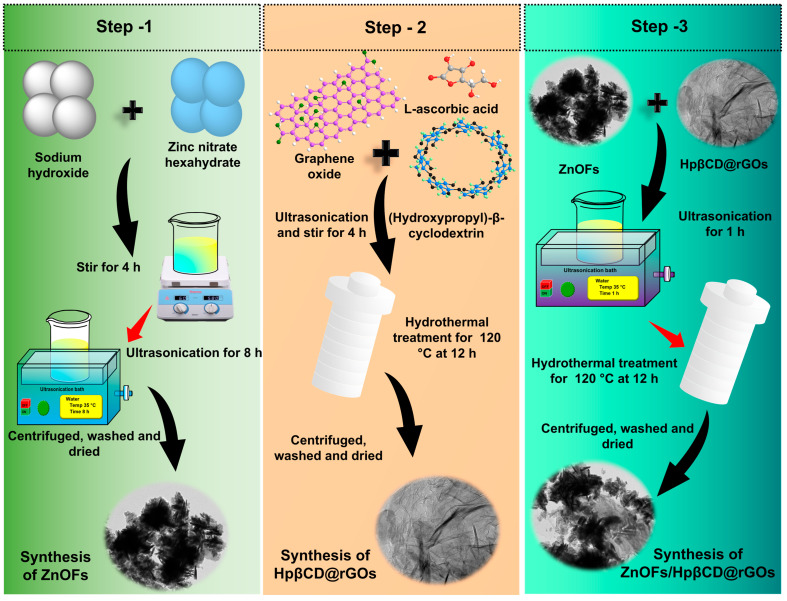
Preparation schemes of ZnOFs, HpβCD@rGOs, and ZnOFs/HpβCD@rGOs.

The ZnOFs and HpβCD@rGOs were combined using sonication and hydrothermal preparation. A solution containing equal amounts of ZnOFs and HpβCD@rGOs was prepared by dissolving them in distilled water. The solution was then subjected to ultrasound treatment with a frequency of around 100 Hz for 1 h. The white solution, which was well dispersed, was thereafter transferred into a container made of Teflon and kept at a temperature of 120 °C for 12 h. This process indicates the synthesis of a composite material consisting of ZnOFs/HpβCD@rGOs. The product, in the beginning, was rinsed with deionized water and ethanol to eliminate the adhering particles. Ultimately, the material became dry by being put in an oven at a temperature of 50 °C overnight. The resulting product (ZnOFs/HpβCD@rGOs) was utilized for further characterization studies. Scheme 1 depicts the formation process of ZnOFs, HpβCD@rGOs, and ZnOFs/HpβCD@rGOs. Equations (1) and (2) show the reaction mechanism of ZnOFs [42].

Zn (NO_3_)_2_.6H_2_O + 2NaOH → Zn (OH)_2_ + 2NaNO_3_ + 6H_2_O (1)

Zn (OH)_2_ → ZnO + H_2_O (2)

### 2.2. Fabrication of ZnOFs/HpβCD@rGOs/GCE

The composite material ZnOFs/HpβCD@rGO, after characterization analysis, was fabricated over the bare GC electrode for the electrochemical study. Before making any modifications, the unmodified GCE was polished with 0.05 μm alumina powder for 3 min until the surface was smooth and reflective. It was then washed with distilled water, dried out, and kept aside. A solution containing 3 mg of ZnOFs/HpβCD@rGOs was prepared by dispersing it in 1 mL of distilled water and subjecting it to ultrasonication for 30 min with a power and frequency of about 40 kHz. This resulted in ZnOFs/HpβCD@rGOs suspensions with a 3 mg/mL composition. About 6 μL of the suspension was drop-cast onto the electrode surface and named ZnOFs/HpβCD@rGOs/GCE. Also, the same procedure was employed for the fabrication of samples like HpβCD@rGOs, rGOs, and ZnOFs over the bare GCE. In addition, a ZnOFs/HpβCD@rGO modified glassy carbon electrode (GCE) was utilized for all electrochemical analytical studies.

### 2.3. Choice of Materials

A novel approach with the development of a ZnO flaky structure combined with hydroxypropyl-beta-cyclodextrin (HP-β-CD) and reduced graphene oxide (rGO) for multifunctional applications as sensing, catalysis, and energy storage was executed. The ZnO with flaky morphology provides a high surface area, while the inclusion of HP-β-CD improves the dispersion and stability of ZnO and rGO, mitigating agglomeration and enabling more efficient interfacial interactions. Furthermore, employing rGO contributes to improved electrical conductivity and mechanical robustness, addressing limitations in conventional ZnO-based systems. By integrating the molecular encapsulation capability of HP-β-CD with the high electron mobility and photoresponse of ZnO and the superior conductivity of rGO, this composite will exhibit unique synergistic properties favoring sensor application. These include enhanced sensitivity and improved charge transfer kinetics, with selective response and excellent electrochemical performances. Higher electrical conductivity and mechanical strength of the composite, along with enhancing the electrochemical performance, provide more active sites for reactions and adsorption. The prevention of HP-β-CD prevents the aggregation of ZnO and rGO, ensuring better dispersion and sustained performance in aqueous and organic environments. Thus, overall, the designed nanocomposite is an excellent candidate for next-generation electronic and optoelectronic devices and is also applicable to the as-developed novel combination [45,46,47,48].

## 3. Results and Discussion

### 3.1. Morphological Studies

**Figure 1:**
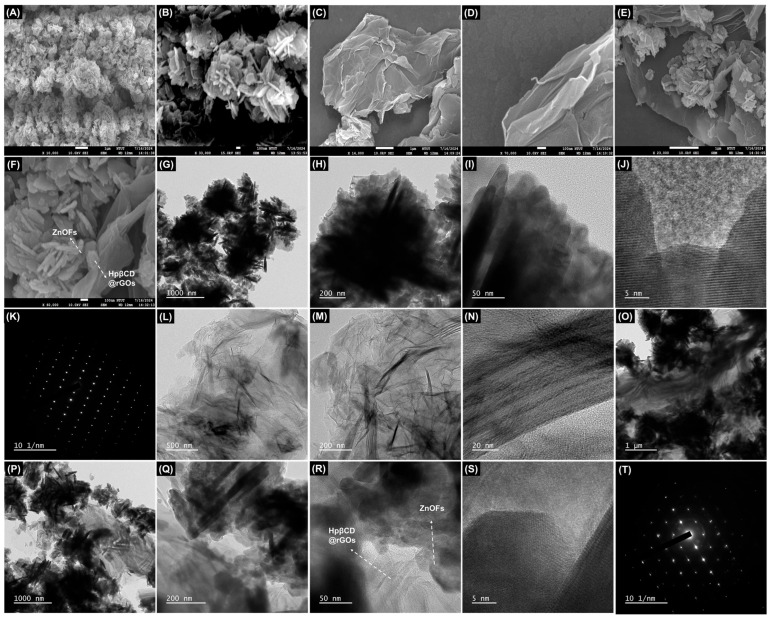
FESEM images of (**A**,**B**) ZnOFs, (**C**,**D**) HpβCD@rGOs, and (**E**,**F**) ZnOFs/HpβCD@rGOs; TEM images of (**G**–**I**) ZnOFs; (**J**) lattice fringes of ZnOFs; (**K**) SAED pattern images of ZnOFs; (**L**–**N**) TEM images of HpβCD@rGOs; (**O**–**R**) TEM images of ZnOFs/HpβCD@rGOs; (**S**) lattice fringes of ZnOFs/HpβCD@rGOs; and (**T**) SEAD pattern images of ZnOFs/HpβCD@rGOs.

Figure 1A–F displays the morphologies of ZnOF, HpβCD@rGO, and ZnOFs/HpβCD@rGO composites as observed from the field Emission Scanning Electron Microscopy (FESEM) analysis. The FESEM morphologies of ultrasonication-treated ZnO structures at different magnifications are shown in Figure 1A,B. The obtained result clearly shows that the prepared ZnO exhibits a flaky structure. These flakes’ structure is flat and regular in shape, as observed in the micro- and nano-graphs obtained at higher and lower magnification. The FESEM images illustrate the stacking, sheet-like, and wrinkled morphology that occurs when HpβCD and reduced graphene oxide sheets (rGOs) (Figure 1C,D) are combined (HpβCD@rGOs). The combined form of ZnOFs and HpβCD@rGOs exhibits a structure that can be like a sandwich, with layers of sheets and flaky structures joined together in a highly interconnected form, as shown in Figure 1E,F. The morphological aspects of ZnOFs, HpβCD@rGOs, and ZnOFs/HpβCD@rGOs composites were examined via TEM and are displayed in Figure 1G–T at different magnifications. The TEM images of ZnOFs followed by FESEM analysis are shown in Figure 1G–I. The presence of several wrinkles in the HpβCD@rGO sheets ensures its two-dimensional nature, as depicted in Figure 1L–N. The transmission electron microscopy images demonstrate that the zinc oxide (ZnOFs) is evenly distributed and intertwined with the HpβCD@rGOs, as illustrated in Figure 1O–R. However, the ZnOFs are strongly bound to the surface of HpβCD@rGOs without any dispersion of zinc oxide, indicating the presence of strong connections between ZnOFs and HpβCD@rGOs. Figure 1J is a high-resolution (HR-TEM) image showing a lattice fringe of ZnOFs, and Figure 1S shows an HR-TEM lattice fringe image of ZnOFs/HpβCD@rGOs. Figure 1K,T displays an SAED pattern that aligns with the results of both XRD analyses of the samples of ZnOFs and ZnOFs/HpβCD@rGOs. The stoichiometry of the elements present in the sample ZnOFs/HpβCD@rGOs was examined via elemental mapping and an EDAX spectrum with TEM analysis, as shown in Figure S2A–E. The chemical mapping and EDAX spectrum analysis confirm the existence of zinc, oxygen, and carbon in the sample. The obtained outcome firmly demonstrates that the composition of the ZnOFs/HpβCD@rGOs combinations is exceptionally pure. Therefore, the electrocatalytic activity for MTN oxidation will be investigated further with all the samples.

**Figure 2:**
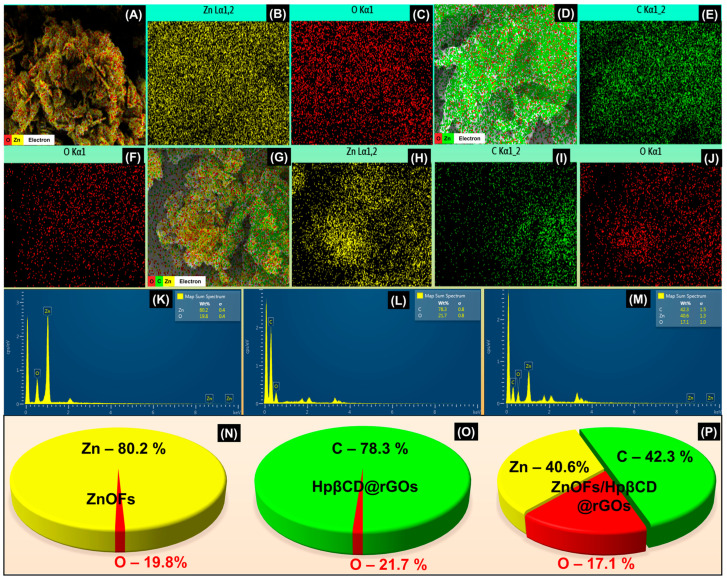
FESEM elemental mapping images of ZnOFs (**A**–**C**), mixed image (**A**), zinc (**B**), oxygen (**C**); HpβCD@rGOs (**D**–**F**), mixed image (**D**), carbon (**G**), oxygen (**F**); ZnOFs/HpβCD@rGOs (**G**–**J**), mixed image (**G**), zinc (**H**), carbon (**I**), oxygen (**J**); EDAX spectrum of ZnOFs (**K**); HpβCD@rGOs (**L**); ZnOFs/HpβCD@rGOs (**M**); and pie chart images of ZnOFs (**N**), HpβCD@rGOs (**O**), ZnOFs/HpβCD@rGOs (**P**).

The elemental mapping and the EDAX spectrum for the samples of ZnOFs, HpβCD@rGOs, and ZnOFs/HpβCD@rGOs were studied and are shown in Figure 2A–P. Figure 2A–C represents the ZnOFs with a mix of Zn and O (zinc and oxygen) elements as separate elemental analysis images. Likewise, HpβCD@rGOs are shown in Figure 2D–F with the elements carbon, oxygen, and their mixture. Figure 2G–J illustrate the presence of zinc, carbon, oxygen, and their mixture, representing the composition of all the elements present in the composite sample ZnOFs/HpβCD@rGOs. Figure 2K–M represent the EDAX spectrum of ZnOFs, HpβCD@rGOs, and ZnOFs/HpβCD@rGOs, while a pie chart showing all the weight ratios of elements of samples of ZnOFs, HpβCD@rGOs, and ZnOFs/HpβCD@rGOs is displayed in Figure 2N–P.

### 3.2. XRD and XPS Analysis

**Figure 3:**
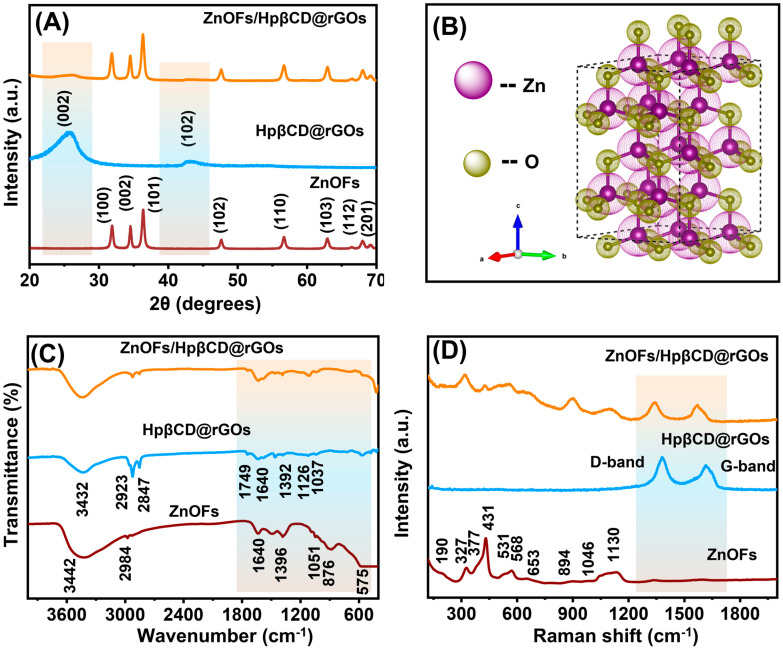
(**A**) XRD spectra of ZnOFs (brown), HpβCD@rGOs (blue), and ZnOFs/HpβCD@rGOs (orange); (**B**) crystal structure of ZnOFs, (**C**) FTIR spectra of ZnOFs (brown), HpβCD@rGOs (blue), and ZnOFs/HpβCD@rGOs (orange); and (**D**) Raman spectra of ZnOFs (brown), HpβCD@rGOs (blue), and ZnOFs/HpβCD@rGOs (orange).

The prepared materials of ZnOFs, HpβCD@rGOs, and ZnOFs/HpβCD@rGOs were investigated with XRD for structural investigation. The corresponding graphs are shown in Figure 3A. The ZnOFs strongly resemble the standard pattern number 01-075-0576, with a hexagonal crystal structure, a space group of P63mc, and space group 186. The lattice parameters are a = 3.2425 Å, b = 3.2425 Å, and c = 5.1946 Å, with alpha(α) = beta (β), and gamma (γ) values as 90.0, 90.0, and 120.0. The occurrence of HpβCD@rGOs was confirmed by identifying these plane values (002) and (102), corresponding to theta values of 26.1° and 42.6°, respectively [40,49,50]. The crystalline and unit cell structures of zinc oxide were determined based on the obtained value, as seen in Figure 3B. The ZnOFs crystal exhibited diffraction peaks at certain two theta values: 31.9°, 34.5°, 36.3°, 47.6°, 56.7°, 62.9°, 66.4°, 68.1°, and 69.1°. These peaks corresponded to the crystalline planes (100), (002), (101), (102), (110), (103), (112), and (201) [19,44]. The XRD data indicate convincing evidence for the increased formation of ZnOFs/HpβCD@rGOs, as well as the absence of other impurities. The peaks exhibit a considerable percentage of crystal formation in the material. The XRD examination revealed the presence of ZnOFs, HpβCD@rGOs, and ZnOFs/HpβCD@rGOs. The size of the crystal was determined using Scherer’s formula (Equation (3)):

(3) $$D=\frac{K\;\lambda }{\beta \;Cos\theta }$$

The variables in the equation are as follows: D indicates the dimension of the crystal, K (0.94) denotes the fixed value of the lattice, λ refers to the wavelengths of X-rays (1.54 Å), β denotes the full-width half maximum of the areas of a high-intensity plane (FWHM), and θ is the angle of diffraction. The overall mean crystal size of each of the prepared samples, ZnOFs, HpβCD@rGOs, and ZnOFs/HpβCD@rGOs, was 50.9 nm, 34.8 nm, and 43.5 nm, respectively.

The XPS analysis for the composite sample ZnOFs/HpβCD@rGOs is shown in Figure S1. From the survey spectrum (Figure S1A), the presence of all the elements that correspond to the ZnOFs/HpβCD@rGO sample was confirmed with the specific peaks raised at the particular binding energies. Figure S1B represents the peak fitting for Zn 2p with two spin–orbit couplings at binding energies 1021 and 1045 eV, representing Zn 2p_3/2_ and Zn 3p_1/2_ [51]. In Figure S1C, the corresponding C 1s peak fitting that is attributed to the presence of carbon is deconvoluted with peaks at 282.8, 284.9, 286.1, and 288.3 eV. These BEs are associated with C-C/C=C, C-O, C=O, and O-C=O bondings in the samples [40], while the presence of oxygen (O 1s) is deconvoluted into three peak fittings at BEs 529.5, 530.1, and 532.6 eV, as observed in Figure S1D. Those peaks are attributed to M-O, C=O, and O-H [40,51]. Thus, from the XPS analysis, the elemental presence of the sample with its bonding is confirmed.

### 3.3. FTIR

The FTIR spectra of ZnOFs, HpβCD@rGOs, and ZnOFs/HpβCD@rGOs samples were determined within the frequency range from 4000–400 cm^−1^, as shown in Figure 3C. The peak observed at 3432 cm^−1^ in the spectra of HpβCD@rGO sheets corresponds to the stretching vibration associated with the hydroxyl (O-H) group, which is wide and intense. In addition, the presence of a carboxyl (C=O) 1640 cm^−1^, epoxy (C–O) 1126 cm^−1^, alkoxy (C–O) 1037 cm^−1^, and (C=O) 1749 cm^−1^ groups located at the edges of these rGO sheets is also shown by the band. The spectra of HpβCD@rGOs reveal original spikes at 2923 cm^−1^ and 2847 cm^−1^, which can be attributed to the asymmetrical and symmetrical stretching vibrations of alkyl groups that occur in the HpβCD moiety of HpβCD@rGOs. The occurrence of the bending vibrations of CH_3_ is confirmed by the typical peaks observed at 1392 cm^−1^, providing further evidence for an excellent chemical functionalization process. The presence of alkyl groups in the Fourier transform infrared spectrum provides a clear indication that the HpβCD compounds have been effectively attached to the surface of rGOs [38,39,52].

The wavenumbers of ZnOFs include 575, 876, 1051, 1396, 1640, 2380, 2984, and 3442 cm^−1^. The dominant absorption peak at 575 cm^−1^ is ascribed to the Zn–O vibration connection between them, indicating evidence for the creation of ZnOFs. At the same time, the broadest absorption band, noticed at 3442 cm^−1^, is associated with the vibration of the O–H bond, indicating the presence of a modest amount of water that was adsorbed on ZnOFs. The band observed at a wavenumber of 1640 cm^−1^ correlates to the bending vibration that occurs due to the O–H bond. The presence of asymmetrical C–H bonds is responsible for the absorption band observed at 2984 cm^−1^. The absorption spectrum around 2380 cm^−1^ is a result of the carbon dioxide (CO_2_) molecules present in the atmosphere. The tiny vibrations recorded at around 876, 1051, and 1396 cm^−1^ are associated with the elongation of the CO bond and the elongation vibration corresponding to the (NH)–CO group, along with the aromatic stretching vibration of –CH, accordingly [53,54,55]. Thus, the ZnO flaky structures and HpβCD@rGOs were successfully combined to form the ZnOFs/HpβCD@rGOs composite material, which involves all of the relevant functional groups.

### 3.4. Raman

Raman spectroscopy is a crucial and responsive analytical instrument for investigating the formation of crystals, structural disorders, and defects in micro-nanostructured materials. Figure 3D depicts the Raman spectra of ZnOFs, HpβCD@rGOs, and ZnOFs/HpβCD@rGOs. The observed spectrum allows for a better understanding of the components of HpβCD@rGOs. The Raman spectra of HpβCD@rGOs exhibit two distinct bands, denoted as G and D. The G band in reduced graphene oxide (rGOs) is characterized by a wide spectral range centered at approximately 1616.3 cm^−1^, while the D band is observed at around 1379.3 cm^−1^. The D band is linked to the A_1g_ mode, which mostly occurs due to defects and edges in graphite compounds. On the other hand, the G band is associated with the symmetrical E_2g_ mode, which comes from the stretching motions of in-plane sp^2^ carbon−carbon double bonding [31,56]. In the case of an ideal ZnOF crystalline material, only the optical phonons located at the Γ point of the Brillouin region are involved in the first-order equations of Raman scattering. Equation (4) describes the presence of optical modes for wurtzite ZnOFs, as predicted by the group theory.

Γ_opt_ = A_1_ + 2B_1_ + E_1_ + 2E_2_ (4)

Each A_1_ and E_1_ mode is a polar branch that is divided into LO (longitudinal optical) and TO (transverse optical) sections. These elements exhibit distinct frequencies because of the macroscopic electrical fields related to the LO phonons. The A_1_, E_1_, and E_2_ modes exhibit a first-order Raman activity. The Raman selection rule dictates that the B_1_ modes are typically inactive in their Raman spectra and are referred to as silent modes. The main phonon modes for hexagonal ZnOFs have been determined at Raman shifts of 377 cm^−1^, 431 cm^−1^, and 568 cm^−1^, relating to the A_1_(TO), E_2H_, and A_1_(LO)/E_1_(LO) modes, correspondingly. A second-order phonon mode, with a Raman shift of around 190 cm^−1^, is attributed to the 2E_2L_ order. The multi-phonon scattering modes are observed at Raman shifts of 327 cm^−1^, 531 cm^−1^, 653 cm^−1^, 894 cm^−1,^ and 1046 cm^−1^. These Raman shift correspond to the 3E_2H_-E_2L_, 2E_1_, E_1_(TO)+E_2L_, 2(E_2H_-E_2L_), and A_1_(TO)+E_1_(TO)+E_2L_ modes, accordingly. Furthermore, the acoustic integration of A_1_ and E_2_ can be recorded at approximately 1130 cm^−1^ [57,58,59]. The composite material of Raman spectra, ZnOFs/HpβCD@rGOs, exhibits band peaks that are similar to those of ZnOFs but with lower intensity. The decrease in intensity may be attributed to the enhanced prominence of defects resulting from the conjugation of HpβCD@rGOs. The presence of peaks at 1616.3 cm^−1^ and 1379.3 cm^−1^ in HpβCD@rGOs confirms the effective development of the composite.

## 4. Electrochemical Studies

### 4.1. Electrochemical Impedance Spectroscopy

**Figure 4:**
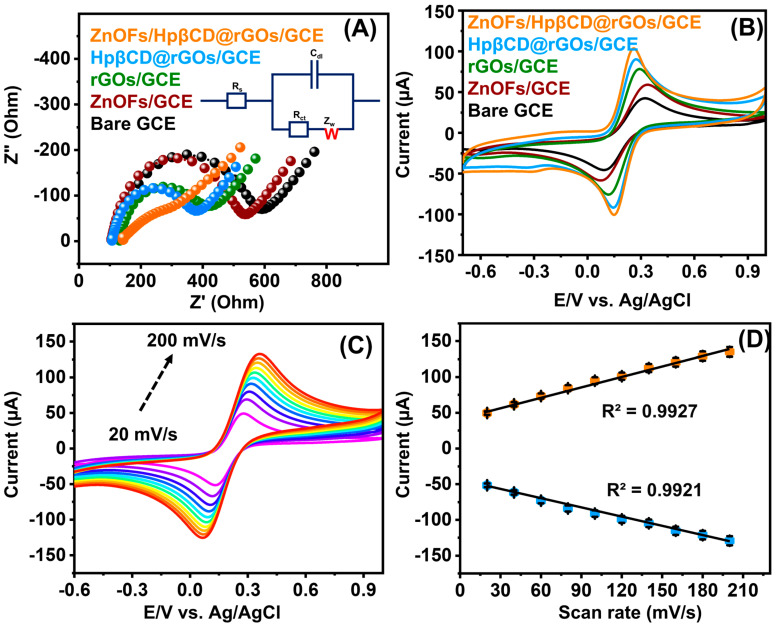
(**A**) EIS spectrum of bare GCE, ZnOFs/GCE, HpβCD@rGOs/GCE, and ZnOFs/HpβCD@rGOs/GCE in an [Fe (CN)_6_]^3−/4−^ (5 mM) and potassium chloride (0.1 M) electrolyte solution with 1 Hz to 100 kHz. (**B**) Cyclic voltammetry spectra of bare GCE, ZnOFs/GCE, HpβCD@rGOs/GCE, and ZnOFs/HpβCD@rGOs/GCE in an [Fe (CN)_6_]^3−/4−^ (5 mM) and potassium chloride (0.1 M) electrolyte solution with a scan rate of 50 mV/s. (**C**) CV graph of scan rate analysis (20 mV/s to 200 mV/s) at ZnOFs/HpβCD@rGOs/GCE in an [Fe (CN)_6_]^3−/4−^ (5 mM) and potassium chloride (0.1 M) electrolyte. (**D**) Corresponding linear graph of scan rate vs. current.

The EIS is an important method for determining the kinetics of reactions between electrodes and monitoring differences in the interface between an electrode and an electrolyte that result from alterations to the electrode surfaces. A testing compound indicating KCl (0.1 M) and [Fe (CN)_6_]^3−/4−^ (5 mM) serves to investigate the electro-kinetics. The outcomes acquired from this investigation are most effectively expressed by the Randle circuit (R_s_ [C_dl_-R_ct_-W]), wherein the solution resistance (R_s_) is consistently coupled to the charge transfer resistance (R_ct_). The Warburg resistance (W) connects in parallel to the double-layer capacitance (C_dl_) [41,60]. The measured diameter of the semicircle within the Nyquist plot illustrates the dynamics of the charge transfer at the interface between the electrode and the electrolyte. Figure 4A depicts the EIS of the unmodified glassy carbon electrode (GCE), which demonstrates an increased charge transfer resistance (R_ct_) due to the GCE’s lack of conductivity, resulting in a sluggish electron transfer rate. Following the construction of the bare GCE with Hp-β-CDT/rGOs, the Nyquist semicircle shrinks, possibly due to the reduced resistance of the modified GCE. This facilitates a faster electron transfer rate and indicates lowered impedance. However, the unmodified GCE yielded an Rct measurement of 464.1 Ω, and the ZnOFs/GCE exhibited 432.7 Ω, while the GCE that had been modified with the rGOs/GCE and HpβCD@rGOs exhibited an Rct value of 285.5 and 269.7 Ω. Following the fabrication and analysis with ZnOFs/HpβCD@rGOs/GCE, there was an observed improvement in the rate at which electrons were transferred. This was evidenced by a decrease in the Rct measurement of 165.1 Ω, indicating a lowered resistance in the electron transfer process. The diffusion procedure occurring between the solutions and the electrode surface is hindered, as indicated by these lowest Rct values. Thus, the ZnOFs/HpβCD@rGOs/GCE nanocomposite was considered appropriate for subsequent electrochemical experiments and the identification of MTN. The findings suggest that the development of the electrode using the ZnOFs/HpβCD@rGOs nanocomposite is very conductive, effectively reducing the resistance across the oxidation investigation and the GCE.

A study was carried out to examine the performance of ZnOFs/HpβCD@rGOs on a fabricated GC electrode. Figure 4B depicts the CV study conducted in the ferricyanide solutions for different modified electrodes like ZnOFs, rGOs, HpβCD@rGOs, and H ZnOFs/HpβCD@rGOs. The measurements were conducted in a solution of 0.1 M KCl (potassium chloride) and with the presence of 5 mM [Fe (CN)_6_]^3−/4−^ (ferricyanide/ferrocyanide) at a scanning rate of 50 mV/s. The observed peak current responses for the various altered GCE electrodes are as follows: bare (Ipa: 42.7), ZnOFs (Ipa: 59.6), rGOs (Ipa: 78.4), HpβCD@rGOs (Ipa: 90.5), and ZnOFs/HpβCD@rGOs nanocomposite (Ipa: 103.9). The increase in the maximum current at ZnOFs/HpβCD@rGOs/GCE is attributed to the contact between ZnOFs and HpβCD@rGOs, as well as the larger active surface area, which enhances the redox reactions mechanism of [Fe (CN)_6_]^3−/4−^. Figure 4C shows the range of scan rates achieved for the ZnOFs/HpβCD@rGOs/GCE nanocomposite, ranging from 20 mV/s to 200 mV/s, in the presence of [Fe (CN)_6_]^3−/4−^. Figure 4D shows a linear plot that represents the anodic and cathodic current as a function of the scanning rate. The results of the linear regression equations for the anodic and cathodic currents are Ipa = 0.4753x + 43.948 (with R^2^ = 0.9927) and Ipc = −0.4231x − 46.573 (with R^2^ = 0.9921), respectively. The active surface area of ZnOFs/HpβCD@rGOs/GCE is considerable in comparison to other modified electrodes, as determined by CV analysis. The Randle–Sevcik formula is used to determine the electroactive surface area (A). This is given by Equation (5) [61]:

I_p_ = (2.69 × 10^5^) n^3/2^AD^1/2^Cʋ^1/2^ (5)

The electroactive surface area of the ZnOFs/HpβCD@rGOs/GCE nanocomposite-modified electrodes was determined to be 0.101 cm^2^ using the given equation. The active surface areas of the bare GCE (0.071 cm^2^) and ZnOFs/GCE (0.078 cm^2^) were calculated. These values were found to be lower than the active surface areas of rGOs/GCE and HpβCD@rGOs/GCE (0.083 cm^2^ and 0.092 cm^2^). The outcomes obtained corresponded with the current peaks observed for each modified electrode, as depicted in Figure 4B. The observed change in the determined outcomes demonstrates the direct relationship between the electrical conductivity and the active surface area, as a result of increased charge transfer.

### 4.2. Electrochemical Detection of MTN with Modified Electrodes, Scan Rate, and pH Performance

**Figure 5:**
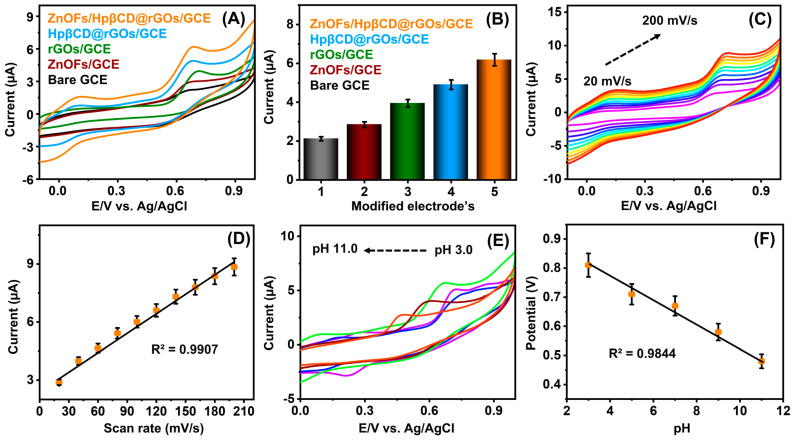
(**A**) shows the cyclic voltammetry signals of different electrodes bare GCE, ZnOFs/GCE, HpβCD@rGOs/GCE, and ZnOFs/HpβCD@rGOs/GCE with the addition of MTN (100 μM) in the presence of a 0.1 M PB solution at a sweep rate of 50 mV/s. (**B**) presents histogram plots comparing the modified electrodes based on their current values, (**C**) displays the CV images of ZnOFs/HpβCD@rGOs/GCE with the addition of 100 μM MTN and a 0.1 M PB solution with scan rates ranging from 20 mV/s to 200 mV/s, (**D**) shows a linear plot of the current values at various scan rates, (**E**) illustrates the CV curves of ZnOFs/HpβCD@rGOs/GCE at different pH levels (ranging from 3.0 to 11.0) with a 100 μM MTN concentration conducted in a 0.1 M PB solution with a scan rate of 50 mV/s, and (**F**) presents the corresponding linear plot of the potential values for different pH levels.

**Scheme 2:**
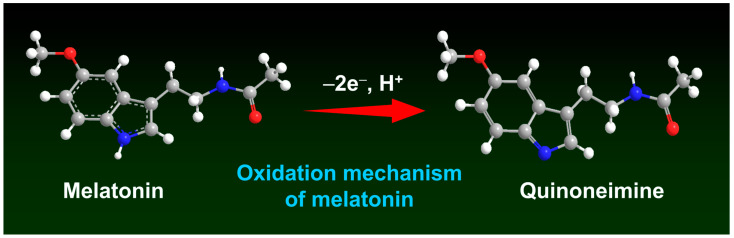
MTN oxidation mechanism.

The electrodes’ bare GCE, ZnOFs/GCE, rGOs/GCE, and HpβCD@rGOs/GCE were initially studied with the CV technique without the presence of MTN to know the difference after the addition of MTN. Figure S3A represents the CV curves of all the modified and unmodified electrodes’ performance at a scan rate of 50 mV/s without the addition of MTN. There were no oxidation response peaks observed for any of the electrodes studied under 0.1 M of the PB solution. The present electrochemical properties of the fabricated electrodes were evaluated using cyclic voltammetry (CV) under the conditions of pH 7.0, a scanning rate of 50 mV/s, and a concentration of 100 μM MTN. The results are presented in Figure 5A in comparison with unfabricated GCE, ZnOFs/GCE, rGOs/GCE, and HpβCD@rGOs/GCE. The unfabricated GCE exhibited a low performance in terms of the oxidation current, measuring 2.2 μA, with a peak potential (Epc) of 0.66 V. In comparison, the ZnOFs/GCE, rGOs/GCE, and HpβCD@rGOs/GCE demonstrated superior but slightly smaller overpotentials of 0.67 V, 0.70 V, and 0.67 V, respectively. Further, the anodic values for ZnOFs/GCE, rGOs/GCE, and HpβCD@rGOs/GCE were measured at 2.8 μA, 3.9 μA, and 4.9 μA, respectively. The ZnOFs/HpβCD@rGOs/GCE demonstrated a clear oxidation peak potential with a notably high peak current of 6.18 μA at 0.68 V. The observed change can be attributed to the increased mobility of electrons toward the surface of the electrodes, which leads to the electrocatalytic oxidation of MTN. The increase in electrocatalytic performance resulted from the binding of HpβCD@rGOs to the ZnOFs, which improved both the surface area as well as the conductivity of electricity. Figure 5B displays a bar graph illustrating the highest level of the current responses observed in the altered electrodes, as stated before. Thus, the ZnOFs/HpβCD@rGOs/GC electrode showed superior electrocatalytic performance in determining MTN when compared with various other electrodes. The electrochemical oxidation of MTN relies on various scanning rates. Figure 5C illustrates the cyclic voltammetry (CV) profiles obtained when a concentration of 100 µM MTN was injected into the phosphate-buffered solution with a pH of 7.0. The scanning rates ranged from 20 mV/s to 200 mV/s. Moreover, the electrical current around the peak of MTN oxidation exhibited a constant rise as the scanning rate increased. Furthermore, as depicted in Figure 5D, the line graph illustrates the relationship between the scanning rate and the oxidation’s peak currents. The formula for a linear regression study was y = 0.0322x + 2.6453, and the corresponding correlation coefficient R^2^ = 0.9907. This finding verifies that the oxidation reaction of MTN on ZnOFs/HpβCD@rGOs/GCE is a mechanism controlled by diffusion. Scheme 2 illustrates the process of the electrochemical oxidation of MTN, where an equal number of electrons are exchanged to produce quinone imine [16,17]. The outcome of this oxidation mechanism is examined using the CV curves produced in the previous investigation.

To determine the optimal electrolyte pH for MTN determination, a cyclic voltammetry (CV) experiment was conducted on the ZnOFs/HpβCD@rGOs/GCE. The experiment involved varying the pH level of the electrolyte solution between 3.0 to 11.0 while maintaining a concentration of 100 μM MTN and a scanning rate of 50 mV/s. The data collected are displayed in Figure 5E,F, suggesting that pH 7.0 serves as the optimal pH buffer for MTN. This is evidenced by the high electrical current responses and the noticed peak shift. The process of protonation and deprotonation responses occurred at pH values ranging from 3.0 to 5.0 and from 9.0 to 11.0. The slower electrochemical interactions with the surface modification and MTN were triggered by the increased acidic and basic media of the electrolytes, resulting in a gradual decrease in the anodic current. The pH was set at an unchanged level of 7.0, resulting in better results compared with previous research. The resultant CV peak values of the study are displayed in Figure 5E. The regression formula obtained for pH optimization was y = −0.0395x + 0.9265, and the correlation coefficient (R^2^) was found to be 0.9844. The derived value of 39 mV according to the linear values is equivalent to the Nernst value of 59 mV [61]. A linear plot representing the relationship between the acquired values of pH vs. potential is displayed in Figure 5F. The pH of 7.0 was maintained at a consistent level throughout the other analyses conducted.

### 4.3. MTN Concentration Variation in CV and DPV

**Figure 6:**
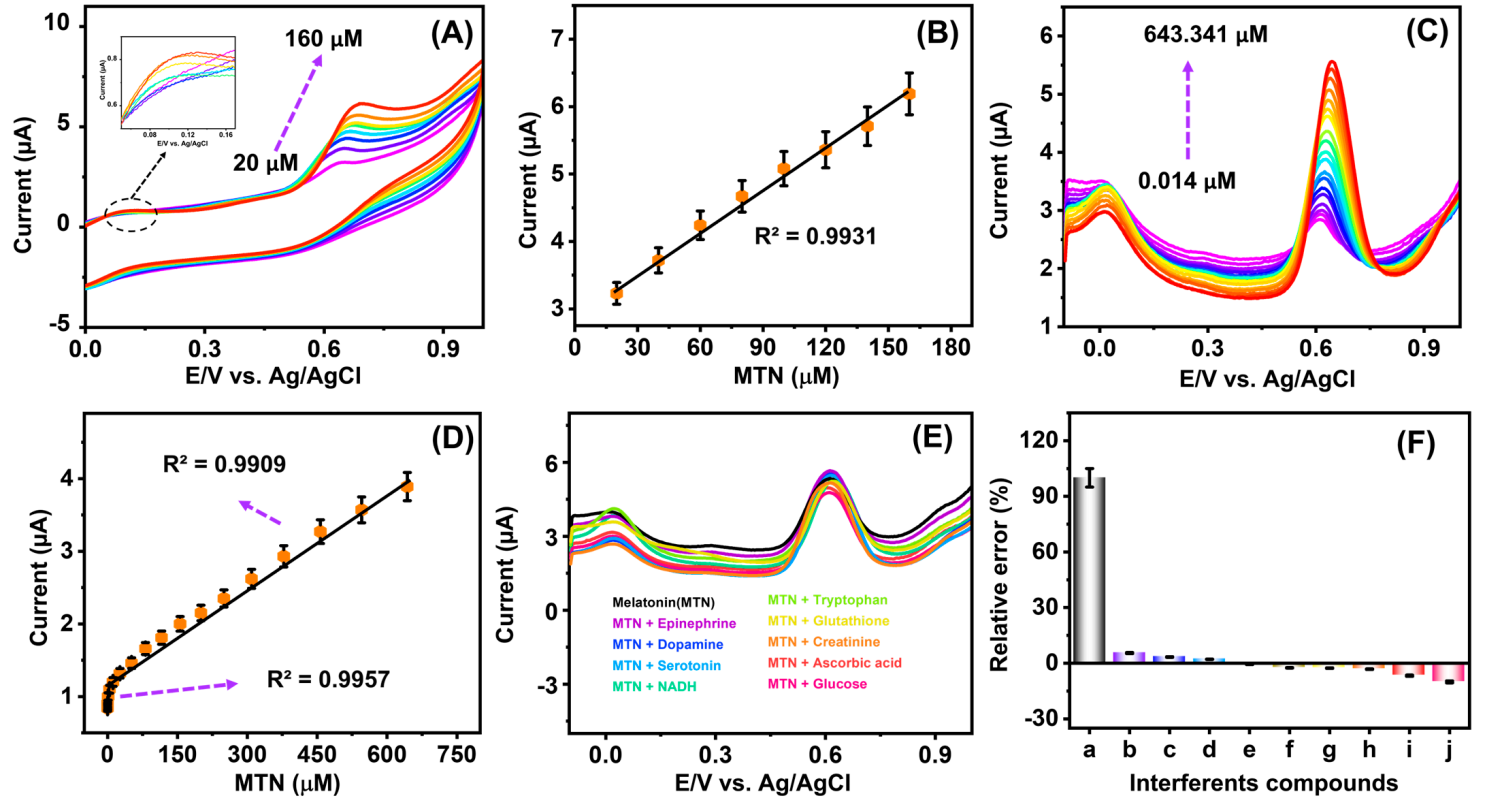
(**A**) displays the cyclic voltammetry images of ZnOFs/HpβCD@rGOs/GCE with different doses (ranging from 20 μM to 160 μM) of MTN conducted using 0.1 M PBS and a scan rate of 50 mV/s. (**B**) A linear plot illustrating the relationship between different concentrations and currents. (**C**) DPV pictures of ZnOFs/HpβCD@rGOs/GCE with varying concentrations of MTN (ranging from 0.014 to 0.149 and 1.149 to 643.341 µM) in a 0.1 M PB solution. (**D**) A calibration plot showing the relationship between the addition of MTN and current. (**E**) displays a selectivity investigation of ZnOFs/HpβCD@rGOs/GCE using 100 μM MTN injection together with different interferents. (**F**) shows the relative error bar histogram plot of the selectivity investigation.

The electrochemical activity of ZnOFs/HpβCD@rGOs/GCE was evaluated by conducting cyclic voltammetry (CV) experiments at different amounts of MTN. Figure 6A and Figure S3B illustrate the CV behavior of ZnOFs/HpβCD@rGOs/GCE when MTN is added in increasing concentrations ranging from 20 µM to 160 µM and 0 to 160 µM. The experiments were conducted in a solution containing phosphate buffer with a pH of 7.0 using a scanning rate of 50 mV/s. The oxidation peak electrical current exhibited a sharp and equal spike in reaction to the amount of MTN. Figure 6B shows a linear relationship between the MTN levels and the oxidation peak current responses, with a regression equation y = 0.0205x + 2.93 and R^2^ of 0.9931. The outcome demonstrated the outstanding electrocatalytic activity of ZnOFs/HpβCD@rGOs/GCE for the oxidation process of MTN.

The DPV technique was used for evaluating MTN at various concentrations in a 0.1 M solution of phosphate buffer utilizing ZnOFs/HpβCD@rGOs/GCE (Figure 6C) and Figure S3C shows the DPV curve of the modified electrode without adding MTN. The oxidation peak current exhibited a steady enhancement as the MTN concentration increased lower to higher continuously from 0.014 to 0.149 µM and from 1.149 to 643.341 µM. Furthermore, as depicted in Figure 6D, there was a linear correlation between Ipa and the amount of MTN. The regression equations for the lower and higher concentrations were y = 0.4335x + 0.8342 and y = 0.0044x + 1.206, with a lower (0.9942) and higher (0.9890) R^2^ value.

Limit of detection (LOD) = 3 σ/S

Sensitivity = Slope/Surface area of the electrode

Moreover, we achieved a wide and continuous linear range of measurements with a detectable limit of 0.0105 µM or 10.5 nM. The sensitivity of the measurements was determined to be 6.19 μA μM^−1^ cm^−2^. In addition, the efficiency of the MTN electrochemical sensing was also compared with the results of several altered electrode components concerning its LOD of MTN, the electrode materials it can detect, and the linear concentration range. The compared outcomes are displayed in Table 1. The outcomes in comparison with other works suggest that the exceptional electrocatalytic performance is a result of the combined effect of ZnOFs and HpβCD@rGOs. Furthermore, the remarkable electrocatalytic ability and conductivity of ZnOFs/HpβCD@rGOs/GCE enhance the process of electrooxidation of MTN on the surface of the electrode.

### 4.4. Selectivity, Repeatability, and Reproducibility Study of ZnOFs/HpβCD@rGOs/GCE by the DPV Method

The MTN sensor’s ability to resist interference was assessed using differential pulse voltammetry (DPV) in the presence of several interference chemicals. Figure 6E illustrates the selectivity analysis performed for the electrochemical oxidation of MTN within an amount of interfering compounds, including serotonin (a), epinephrine (b), dopamine (c), nicotinamide adenine dinucleotide (NADH) (d), tryptophan (e), glutathione (f), creatinine (g), glucose (h), and ascorbic acid (i). The DPV method was investigated in the presence of a PB solution of pH 7.0 and other interference analytes that were five-fold lesser in concentration. Figure 6F illustrates a diagram depicting the relative error percentage histogram of different interfering chemicals. The findings validate that ZnOFs/HpβCD@rGOs/GCE exhibits excellent selectivity towards MTN.

Crucial parameters such as repeatability and reproducibility were studied using cyclic voltammetry, with the inclusion of MTN. The investigations were conducted using similar settings, including 75 μM of MTN, a constant scanning rate of 50 mV/s, and a ZnOFs/HpβCD@rGO-fabricated GCE. The reproducibility study, conducted using the aforementioned parameters, is depicted in Figure S4A. The CV graphs depict the oxidation peaks occurring at the peak potential, with a consistent anodic peak current observed throughout five consecutive investigations. The repeatability research was conducted using the aforementioned conditions for five different repeated experiments. The CV curves produced are shown in Figure S4B. Both the potential and anodic peak current exhibited similar results for identifying MTN. Despite conducting multiple runs of the examination, the reaction to MTN at ZnOFs/HpβCD@rGOs/GCE remained unchanged. Therefore, the electrode exhibited excellent performance in detecting MTN. These histogram diagrams are displayed in Figure S4C,D for the study mentioned previously.

### 4.5. Real Sample Investigation

**Figure 7:**
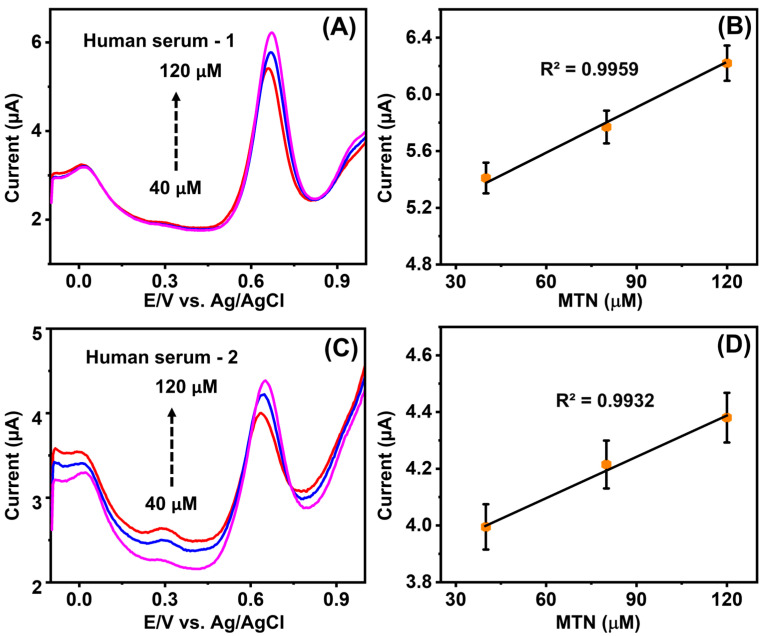
Real sample studies of ZnOFs/HpβCD@rGOs/GCE with the presence of MTN, (**A**) human serum sample-1, and (**C**) human serum sample-2, with their (**B**,**D**) corresponding linear plots.

The real sample monitoring was examined in the human serum sample-1 and human serum sample-2. Each sample underwent pretreatment earlier for analysis, following the procedure outlined below. The human serum sample-1 and human serum sample-2 were initially subjected to centrifugation at a speed of 1600 rotations per minute (rpm) for 20 min. The samples were stored at 4 °C until being taken for analysis, which did not exceed a week. Before analysis, the collected samples were diluted with 0.1 M phosphate buffer and taken directly for analysis. The ZnOFs/HpβCD@rGOs/GCE was examined using real human serum samples, which are free of MTN, with the injection of MTN ranging from 40 to 120 µM following the regular addition method. The pretreated samples exhibited favorable overall recovery outcomes (99% and 98%) characterized by increased peak outcomes. The acquired DPV curves are displayed in Figure 7A,C, and the associated linear plots are presented in Figure 7B,D with the relevant correlation coefficients as the inset. Table S1 depicts the real sample analysis recovery percentage tabulation.

## 5. Conclusions

In conclusion, a crystal-like composite of ZnOFs/HpβCD@rGOs/GCE has been developed by employing an easy sonication method, utilizing presynthesized ZnOF architectures and HpβCD@rGO sheets. The confirmation of the synergistic effect of the component has been established by examinations of its structural and electrical performance. Subsequently, the combination of materials was applied onto a glassy carbon electrode that had been processed initially. This allowed for the specific identification of MTN at a small scale, with an acceptable detection range of 0.014–0.149 and 1.149–643.341 µM. The sensors that were created also showed an excellent response through repeatability and reproducibility studies. The detection using differential pulse voltammetry (DPV) analysis was carried out by the oxidation of MTN. Subsequently, the technique of sensing was utilized for testing actual serum from human samples, revealing the acceptable recovery range of the composite component that was constructed. This indicates its suitability for a variety of applications.

## Figures and Tables

**Table 1:** This table presents a comparison of various modified electrodes used for the monitoring of MTN in relation to previously published publications.

Electrode Modified Materials	MTN Sensing Techniques	MTN Wide Linear Range (µM)	MTN Limit of Detection (µM)	Ref.
^1^ CDE	CV	0.0025–1.1 (mmol/L)	1.3	[13]
^2^ GPH-CSPE	Amperometric (IT)	1–300	0.87	[62]
^3^ VMSF/HErGO-CNT/ITO	DPV	20–100	0.014	[6]
^4^ CNTs and graphene-based CSPE	DPV	0.005–3mM	1.1	[63]
^5^ Fe_3_O_4_@Cu (Im)_2_/GSPE	DPV	1.0–300.0	0.24	[14]
^6^ MIP/Cu_x_O/MXene/SPCE	DPV	5–700	0.029	[64]
^7^ PTBO/MWCNTs/GCE	DPV	1–1000	0.027	[65]
ZnOFs/HpβCD@rGOs/GCE	DPV	0.014–0.149 and 1.149–643.341 µM	0.0105 µM or 10.5 nM	This work

DPV—differential pulse voltammetry; CV—cyclic voltammetry; ZnOFs/HpβCD@rGOs/GCE—zinc oxide flaky structures/hydroxypropyl-beta-cyclodextrin functionalized reduced graphene oxide sheets; ^1^ CE—capilary electrophoresis; ^2^ GPH-CSPE—graphene-carbon screen-printed electrode; ^3^ VMSF/HErGO-CNT/ITO—vertically oriented mesoporous silica-nanochannel films (VMSFs) on highly electrochemically reduced graphene-oxide–carbon nanotubes/indium tin oxide; ^4^ CNTs and graphene-based CSPEs—carbon nanotubes and graphene-based carbon screen-printed electrodes; ^5^ Fe_3_O_4_@Cu (Im)_2_/GSPE—magnetite@copper (II) imidazolate nanoparticles/graphite-screen-printed electrode; ^6^ MIP/Cu_x_O/MXene/SPCE—molecularly imprinted/copper oxide/MXerne/screen-printed-carbon electrode; and ^7^ PTBO/MWCNTs/GCE—polytoluidine blue O/multiwall carbon nanotubes/glassy carbon electrode.

## Data Availability

The original contributions presented in this study are included in the article/Supplementary Materials. Further inquiries can be directed to the corresponding author.

## Supplementary Materials

The following supporting information can be downloaded at: https://www.mdpi.com/article/10.3390/s25113266/s1, Figure S1 XPS overall survey scan of ZnOFs/HpβCD@rGOs (A), Zn 2p (B), C 1S (C), and O 1s (D), Figure S2 TEM elemental mapping images of ZnOFs/HpβCD@rGOs (A-D), mix image (A), Zn (B), O (C), C (D), and EDAX spectrum of ZnOFs/HpβCD@rGOs (E), Figure S3 (A) shows the cyclic voltammetry signals of different electrodes: bare GCE, ZnOFs/GCE, HpβCD@rGOs/GCE, and ZnOFs/HpβCD@rGOs/GCE performed without the concentration of MTN in the presence of a 0.1 M PB solution at a sweep rate of 50 mV/s, (B) displays the cyclic voltammetry images of ZnOFs/HpβCD@rGOs/GCE with different doses (ranging from 0 μM to 160 μM) of MTN conducted using 0.1 M PBS and a scan rate of 50 mV/s, and (C) DPV curve of ZnOFs/HpβCD@rGOs/GCE without the addition of MTN in a 0.1 M PB solution, Figure S4 CV profiles of ZnOFs/HpβCD@rGOs/GCE with 75 µM of MTN (A) reproducibility, (B) repeatability, and (C and D) its corresponding histogram diagrams of reproducibility and repeatability, Table S1. Real sample analysis recovery percentage tabulation (n = 3).
