# Supplementary material for: A Zinc Oxide Interconnected Hydroxypropyl-Beta-Cyclodextrin/rGO Nanocomposite as an Electrocatalyst for Melatonin Detection: An Ultra-Sensitive Electrochemical Sensor
Source: Sensors (Basel). 2025 May 22;25(11):3266. doi: 10.3390/s25113266 (PMC12157216; doi:10.3390/s25113266)
Supplement: Supplementary file 1 [file sensors-25-03266-s001.zip › sensors-3562013-supplementary.pdf]

sensors

Article

A Zinc Oxide Interconnected Hydroxypropyl-Beta-Cyclodextrin/rGO Nanocomposite as an Electrocatalyst for Melatonin Detection: An Ultra-Sensitive Electrochemical Sensor

Kuo-Yuan Hwa [1,2,*], Aravindan Santhan [1,2], Chun-Wei Ou [2] and Cheng-Han Wang [2]

[1] Department of Molecular Science and Engineering, National Taipei University of Technology, Taipei 10608, Taiwan; aravindan@mail.ntut.edu.tw
[2] Graduate Institute of Organic and Polymeric Materials, National Taipei University of Technology, Taipei 10608, Taiwan; t112518088@ntut.edu.tw (C.-W.O.); t112518009@ntut.edu.tw (C.-H.W.)
* Correspondence: kyhwa@mail.ntut.edu.tw; Tel.: +02-27712171 (ext. 2419(0), 2439 or 2442)

Academic Editor: Alfredo de la Escosura-Muñiz

Received: 17 March 2025
Revised: 27 April 2025
Accepted: 19 May 2025
Published: 22 May 2025

Citation: Hwa, K.-Y.; Santhan, A.; Ou, C.-W.; Wang, C.-H. A Zinc Oxide Interconnected Hydroxypropyl-Beta-Cyclodextrin/rGO Nanocomposite as an Electrocatalyst for Melatonin detection: An Ultra-Sensitive Electrochemical Sensor. *Sensors* **2025**, *25*, 3266. https://doi.org/10.3390/s25113266

Copyright: © 2025 by the authors. Licensee MDPI, Basel, Switzerland. This article is an open access article distributed under the terms and conditions of the Creative Commons Attribution (CC BY) license (https://creativecommons.org/licenses/by/4.0/).

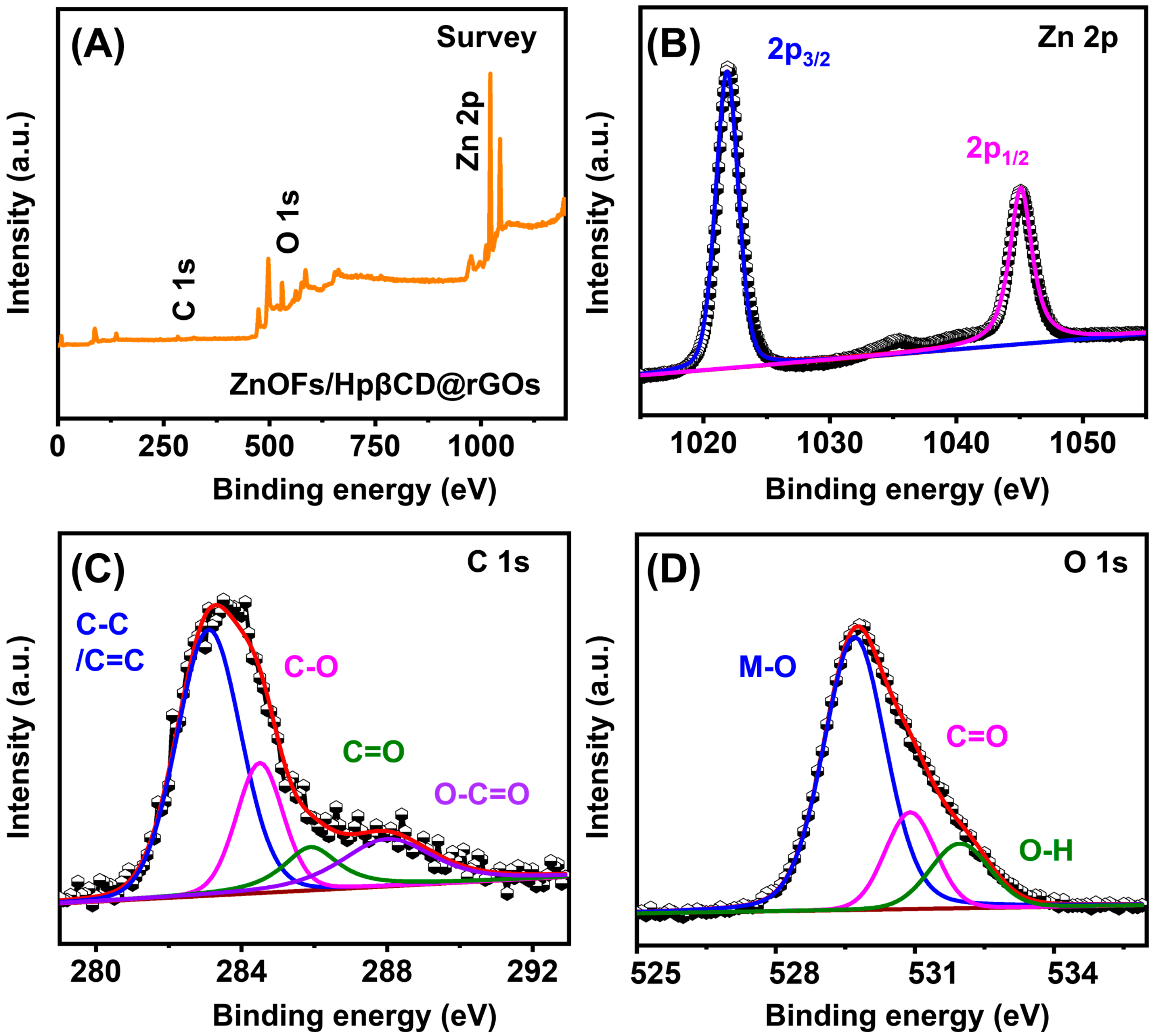

Figure. S1 XPS overall survey scan of ZnOFs/HpβCD@rGOs **(A)**, Zn 2p **(B)**, C 1S **(C)**, and O 1s **(D)**.

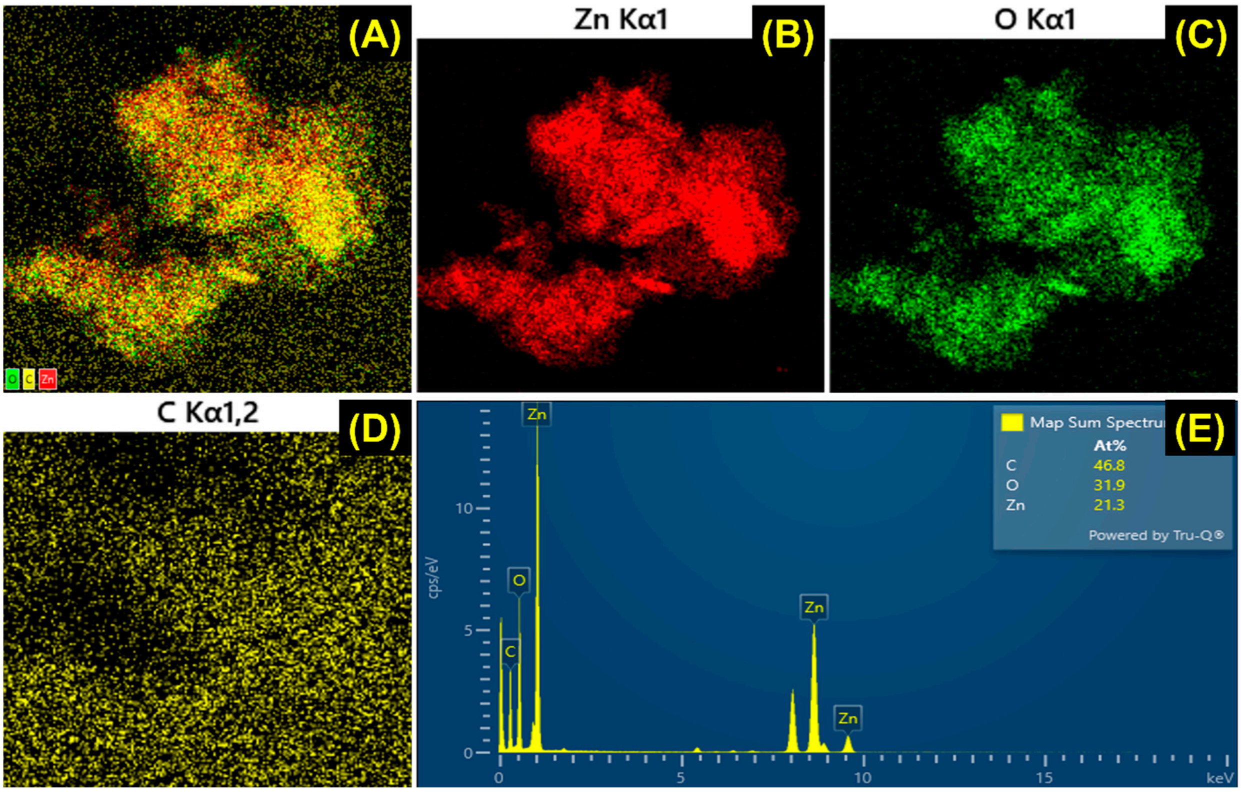

Figure. S2 TEM elemental mapping images of ZnOFs/HpβCD@rGOs **(A-D)**, mix image **(A)**, Zn **(B)**, O **(C)**, C **(D)**, and EDAX spectrum of ZnOFs/HpβCD@rGOs **(E).**

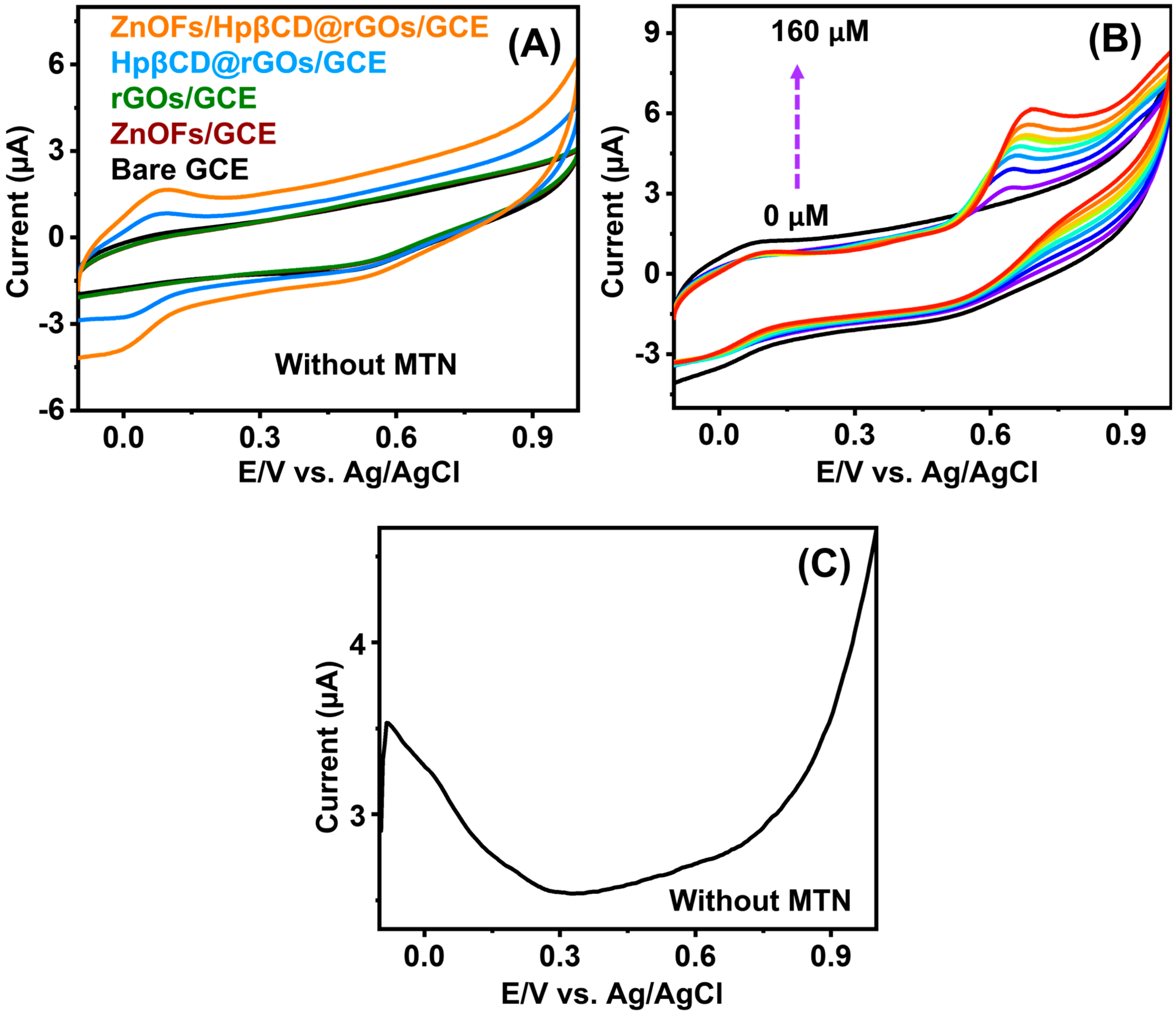

Figure. S3 (A) shows the cyclic voltammetry signals of different electrodes: bare GCE, ZnOFs/GCE, HpβCD@rGOs/GCE, and ZnOFs/HpβCD@rGOs/GCE performed without the concentration of MTN in the presence of a 0.1 M PB solution at a sweep rate of 50 mV/s, **(B)** displays the cyclic voltammetry images of ZnOFs/HpβCD@rGOs/GCE with different doses (ranging from 0 μM to 160 μM) of MTN conducted using 0.1 M PBS and a scan rate of 50 mV/s, and **(C)** DPV curve of ZnOFs/HpβCD@rGOs/GCE without the addition of MTN in a 0.1 M PB solution.

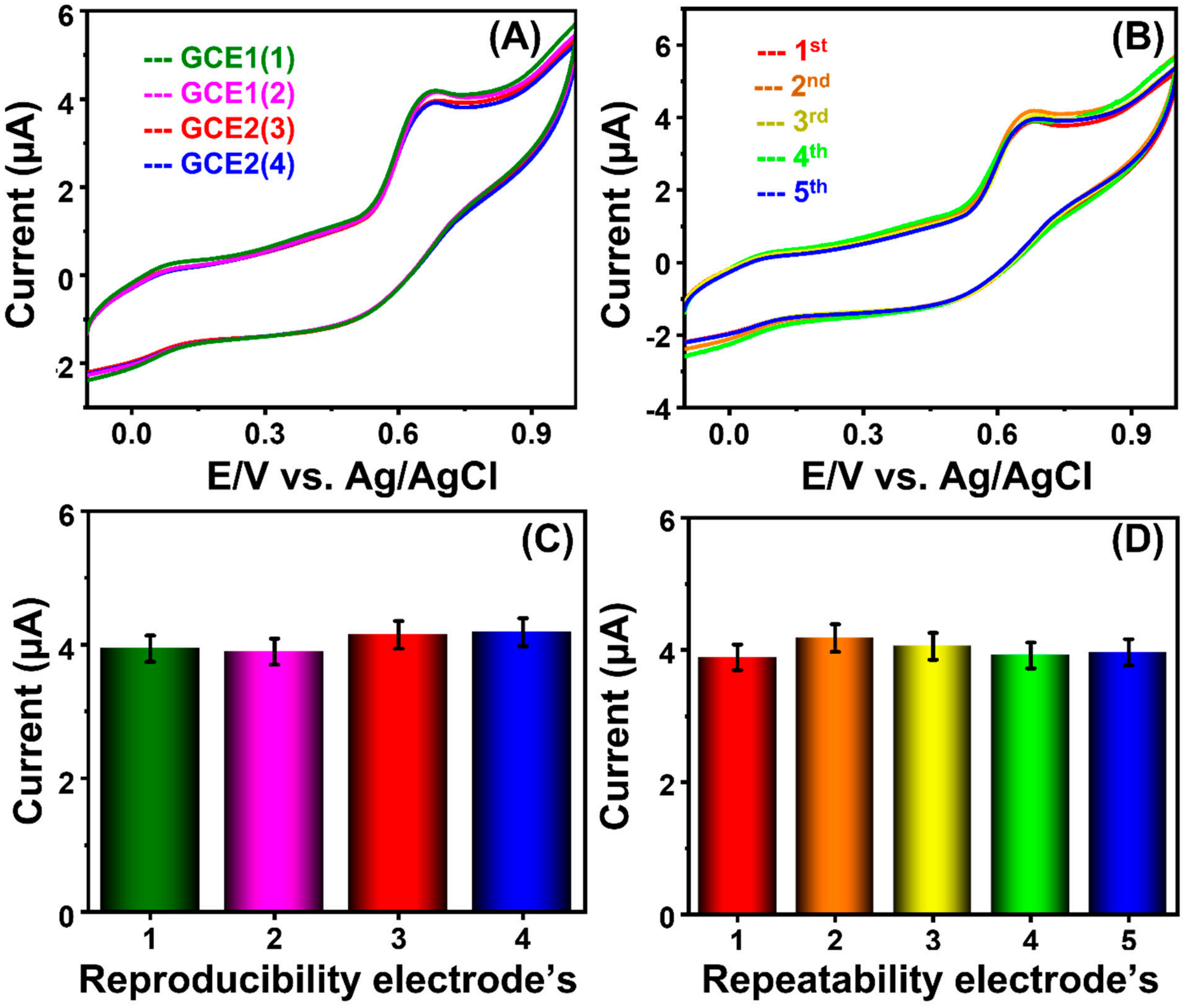

Figure. S4 CV profiles of ZnOFs/HpβCD@rGOs/GCE with 75 µM of MTN **(A)** reproducibility, **(B)** repeatability, and **(C and D)** its corresponding histogram diagrams of reproducibility and repeatability.

Table. S1. Real sample analysis recovery percentage tabulation (n = 3).

Sample	Added (µM)	Detected (µM)<br>--------------------<br>DPV	Detection Rate (%) (Mean ± RSD) (n=3)
Human blood serum-1	0	-	-
	40	39.86	99.65 ± 0.07
	80	79.68	99.60 ± 0.05
	120	119.62	99.68 ± 0.11
Human blood serum-2	0	-	-
	40	39.72	99.30 ± 0.09

	80	79.71	99.63 ± 0.13
	120	119.60	99.66 ± 0.06

Disclaimer/Publisher's Note: The statements, opinions and data contained in all publications are solely those of the individual author(s) and contributor(s) and not of MDPI and/or the editor(s). MDPI and/or the editor(s) disclaim responsibility for any injury to people or property resulting from any ideas, methods, instructions or products referred to in the content.
